# Discovery and validation of biomarkers to aid the development of safe and effective pain therapeutics: challenges and opportunities

**DOI:** 10.1038/s41582-020-0362-2

**Published:** 2020-06-15

**Authors:** Karen D. Davis, Nima Aghaeepour, Andrew H. Ahn, Martin S. Angst, David Borsook, Ashley Brenton, Michael E. Burczynski, Christopher Crean, Robert Edwards, Brice Gaudilliere, Georgene W. Hergenroeder, Michael J. Iadarola, Smriti Iyengar, Yunyun Jiang, Jiang-Ti Kong, Sean Mackey, Carl Y. Saab, Christine N. Sang, Joachim Scholz, Marta Segerdahl, Irene Tracey, Christin Veasley, Jing Wang, Tor D. Wager, Ajay D. Wasan, Mary Ann Pelleymounter

**Affiliations:** 1 Department of Surgery and Institute of Medical Science, University of Toronto, Toronto, ON Canada; 2 Division of Brain, Imaging and Behaviour, Krembil Brain Institute, Toronto Western Hospital, University Health Network, Toronto, ON Canada; 3 Department of Anesthesiology, Perioperative and Pain Medicine, Stanford University School of Medicine, Stanford, CA USA; 4 Teva Pharmaceuticals, Frazer, PA USA; 5 Center for Pain and the Brain, Harvard Medical School, Boston, MA USA; 6 Mycroft Bioanalytics, Salt Lake City, UT USA; 7 Teva Pharmaceutical Industries, Frazer, PA USA; 8 Xyzagen, Pittsboro, NC USA; 9 Pain Management Center, Brigham and Women’s Hospital and Harvard Medical School, Boston, MA USA; 10 The Vivian L. Smith Department of Neurosurgery, The University of Texas Health Science Center at Houston, McGovern Medical School, Houston, TX USA; 11 Department of Perioperative Medicine, Clinical Center, NIH, Rockville, MD USA; 12 Division of Translational Research, National Institute of Neurological Disorders and Stroke, NIH, Rockville, MD USA; 13 The Biostatistics Center, Milken Institute School of Public Health, The George Washington University, Washington, DC USA; 14 Department of Neuroscience and Department of Neurosurgery, Carney Institute for Brain Science, Brown University, Providence, RI USA; 15 Department of Anesthesiology, Brigham and Women’s Hospital and Harvard Medical School, Boston, MA USA; 16 Neurocognitive Disorders, Pain and New Indications, Biogen, Cambridge, MA USA; 17 Asarina Pharma, Copenhagen, Denmark; 18 Nuffield Department of Clinical Neurosciences, University of Oxford, Oxford, UK; 19 Chronic Pain Research Alliance, Bethesda, MD USA; 20 Department of Anesthesiology, Perioperative Care and Pain Medicine, NYU School of Medicine, New York, NY USA; 21 Department of Psychological and Brain Sciences, Dartmouth College, Hanover, NH USA; 22 Anesthesiology and Perioperative Medicine and Psychiatry, University of Pittsburgh, Pittsburgh, PA USA

**Keywords:** Biomarkers, Chronic pain

## Abstract

Pain medication plays an important role in the treatment of acute and chronic pain conditions, but some drugs, opioids in particular, have been overprescribed or prescribed without adequate safeguards, leading to an alarming rise in medication-related overdose deaths. The NIH Helping to End Addiction Long-term (HEAL) Initiative is a trans-agency effort to provide scientific solutions to stem the opioid crisis. One component of the initiative is to support biomarker discovery and rigorous validation in collaboration with industry leaders to accelerate high-quality clinical research into neurotherapeutics and pain. The use of objective biomarkers and clinical trial end points throughout the drug discovery and development process is crucial to help define pathophysiological subsets of pain, evaluate target engagement of new drugs and predict the analgesic efficacy of new drugs. In 2018, the NIH-led Discovery and Validation of Biomarkers to Develop Non-Addictive Therapeutics for Pain workshop convened scientific leaders from academia, industry, government and patient advocacy groups to discuss progress, challenges, gaps and ideas to facilitate the development of biomarkers and end points for pain. The outcomes of this workshop are outlined in this Consensus Statement.

## Abstract (web summary)

In 2018, the Discovery and Validation of Biomarkers to Develop Non-Addictive Therapeutics for Pain workshop convened to discuss strategies to facilitate the development of biomarkers and end points for pain. The outcomes of this workshop are outlined in this Consensus Statement.

## Introduction

Chronic pain, defined as pain that occurs on ≥50% of days over a period of at least 6 months or as pain that persists for at least 3 months^1^, represents one of the most prevalent, costly and disabling health conditions^2^. In the 2017 Global Burden of Disease Study, low back pain and headache disorders ranked in the top three global causes of years lived with disability^3^. A 2018 analysis of Medical Expenditure Panel Survey data found that the proportion of adults in the United States reporting at least one painful health condition increased from 120 million (32.9%) in 1997–1998 to 178 million (41%) in 2013–2014 (ref.^4^). An estimated 50–100 million adults in the United States live with chronic pain that can substantially restrict work, social and self-care activities^5–7^. The increased prevalence of chronic pain has been associated with a substantial rise in the use of prescription opioids to treat non-cancer-related chronic pain, with 275 million people worldwide using opioids in 2016 and 27 million of these people developing opioid use disorders^8^. In addition, each day in the United States, >90 individuals die from opioid overdose^9^. Solutions are urgently needed to address the chronic pain and prescription opioid crises around the world^10^.

In April 2018, the NIH launched the Helping to End Addiction Long-term (HEAL) Initiative to stem the national opioid public health crisis. Experts from public and private organizations identified two major areas that would most benefit from focused efforts by the NIH, alone or in partnership with outside organizations: first, improvement of treatment options for opioid misuse and addiction in adults and for infants exposed to opioids; and, second, enhancement of pain management through non-addictive pharmacological therapeutics and non-pharmacological interventions, as well as improved treatment integration into healthcare systems. To help improve pain management, there is a need to develop translational tools, such as well-validated biomarkers and objective clinical trial end points for pain, which could be used to select participants for clinical trials, demonstrate target engagement of new therapeutics and predict the therapeutic response. This topic provided the basis for the NIH Discovery and Validation of Biomarkers to Develop Non-Addictive Therapeutics for Pain workshop. In this Consensus Statement, we report on the outcomes of this workshop and provide guidance for the development and validation of biomarkers and end points for chronic pain.

## Methods

As a result of the NIH HEAL initiative-sponsored Pain Biomarker Workshop planning discussions, the National Institute of Neurological Disorders and Stroke (NINDS), in collaboration with other NIH institutes — the National Center for Complementary and Integrative Health (NCCIH), the National Institute of Nursing Research (NINR), the National Institute on Drug Abuse (NIDA), the National Cancer Institute (NCI), the National Institute on Alcohol Abuse and Alcoholism (NIAAA), the National Institute of Biomedical Imaging and Bioengineering (NIBIB), the Office of Behavioral and Social Sciences Research (OBSSR) and the National Institute of Dental and Craniofacial Research (NIDCR) — held the Discovery and Validation of Biomarkers to Develop Non-Addictive Therapeutics for Pain workshop on 14–15 November 2018. The goals of the workshop were to evaluate the state of the science in pain biomarker development; to explore potential scientific and collaborative approaches that could facilitate the discovery and validation of robust biomarkers and end points; and to inform the community about regulatory standards and guidelines for the development of biomarkers and end points.

The workshop sessions showcased the status of the science for different categories and modalities of biomarkers or end points, and the potential for transformational improvements in clinical trial design and clinical practice, focused on non-addictive treatment of pain. The five main discussion topics are listed in Box 1. Each session was followed by a panel discussion, in which the audience was encouraged to discuss their views on how the biomarkers presented in the session might be translated into useful tools for pain therapeutic development. The workshop closed with a discussion of the realities of biomarker development and the potential of these markers to transform treatment options for people afflicted with chronic pain.

The workshop co-chairs, Mary Ann Pelleymounter and Simon Tate, obtained feedback from 29 participants who volunteered to write this Consensus Statement. The writing team consisted of 16 academic investigators, 6 scientists from the pharmaceutical industry, the Director of the Chronic Pain Alliance (a patient-based organization) and 3 Program Directors from the NIH. Karen Davis was selected to lead the writing effort with the assistance of the workshop co-chairs. An outline for the summary and a publishing venue was proposed with group consensus. The manuscript was divided into sections, each assigned to a section lead with between two and seven writers. Monthly and ad hoc teleconferences were held to track progress against timelines and content. All sections were integrated into a full manuscript to create this Consensus Statement.

Box 1 Discussion topics The workshop discussion centred on five main topics:

- Topic #1: what are the general guiding principles for biomarker development?
- Topic #2: what is the current state of the science for pain biomarkers and end points?
- Topic #3: what are the challenges in developing and implementing pain biomarker use in clinical trials and clinical practice?
- Topic #4: how are regulatory agencies addressing the need for validated biomarkers?
- Topic #5: what are the societal and ethical issues that should be considered in the development and use of pain biomarkers?

## The need for pain biomarkers

Current pharmacological, interventional, behavioural and surgical therapies for chronic pain have limited efficacy, as reflected in the high prevalence of chronic pain, the low rates of functional recovery and return to work, and the continued reliance on opioid analgesics^5,6,11^. Three new drugs that utilize a non-opioid mechanism of action were recently approved by the FDA for the treatment of migraine^12^, but a large gap still remains in the available armamentarium to treat other types of chronic pain effectively.

There are numerous challenges in the identification and development of safe and effective, non-addictive pain medications. For example, most clinical trials to date have failed owing to a lack of efficacy^13^. Reasons for these failures could include insufficient understanding of the neurobiological mechanisms of chronic pain, poor translation of preclinical data and challenges inherent to clinical trials, such as large placebo responses. Perhaps most importantly, healthcare professionals who treat pain lack reliable biomarkers to demonstrate therapeutic target engagement, to stratify patients and to predict disease progression or therapeutic response^14^. Evidence from other therapeutic areas, such as cardiovascular and metabolic diseases, have illustrated the value of such biomarkers^15^. The importance of target engagement biomarkers was highlighted by AstraZeneca, who reported that clinical proof of mechanism by these biomarkers increased the probability of project advancement to phase II by 25% (ref.^16^). Furthermore, a large biomarker business intelligence analysis of clinical development success rates between 2006 and 2015 showed that availability of selection or stratification biomarkers increased the probability of success by as much as 21% in phase III clinical trials and by as much as 17.5% from phase I to regulatory approval in all disease areas^17^.

**Fig. 1:**
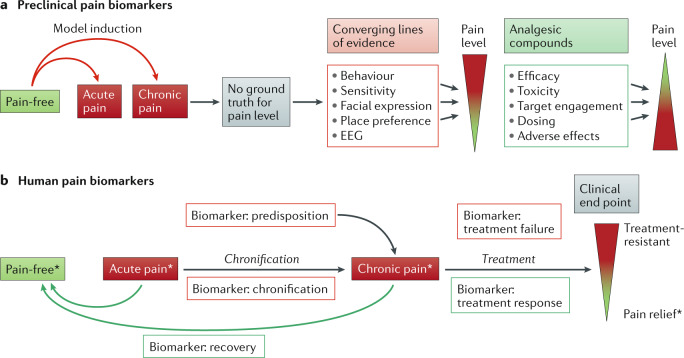
Preclinical and human pain biomarkers. **a** | Development of preclinical pain biomarkers starts with induction of different modalities of pain that are clinically relevant. In the absence of a ground truth for pain in animals, a critical first step relies on converging lines of evidence from behavioural, electrophysiological and other overt signs. The next step is to demonstrate reversal of these signs using analgesic compounds with proven efficacy in humans. **b** | Development of human pain biomarkers starts with the individual’s self-report, also known as the ground truth (asterisks), and a set of signs and symptoms, with the goal of defining objective methods and criteria, as well as end points for assessing, predicting and/or classifying pain and analgesia. Thus, biomarkers are obtained to indicate chronic pain predisposition, pain mechanisms, diagnostic stratification, chronification, recovery and treatment outcome (response or failure).

Patient stratification biomarkers are especially important to inform the design of clinical trials in disease areas with heterogeneous pathophysiology, such as pain. By enabling selection of patients from a mixed disease population, these biomarkers could reduce variability of the response to an intervention and the consequent expense of unnecessarily large clinical trials of pain therapeutics. Patient selection biomarkers also provide the potential for a personalized approach to treat pain, as is used in the field of oncology^18,19^. Biomarkers that represent acute and chronic pain, predisposition to develop chronic pain, and pain chronification, recovery and treatment outcomes are being sought in both preclinical animal models and human studies (Fig. 1).

Rigorously validated biomarkers and end points also have the potential to provide objective measures of pain. Medical conditions are traditionally characterized by signs (objective evidence) and symptoms (subjective reports). Given that pain is a subjective experience, the gold standard for its assessment has long been the individual self-report^20^. Thus, pain as defined by an individual is their ground truth. Accordingly, the International Association for the Study of Pain (IASP) defines pain as “an unpleasant sensory and emotional experience associated with actual or potential tissue damage or described in terms of such damage”^21^. At present, assessment of pain in the clinical setting relies largely on rating scales and symptom-based questionnaires^22–26^. However, these subjective measures are influenced by contextual factors and are only moderately reliable, even with intensive training programmes to improve the ability of individuals to accurately self-report pain^27^. These limitations highlight the lack of universally accepted objective biomarkers for pain and the pressing need for these tools^27^. After rigorous validation, neuroimaging and neurophysiological measurements such as PET, MRI, EEG, quantitative sensory testing (QST)^28–30^ and genetic and genomic analysis could be considered as additional tools to complement self-reports in the assessment and differentiation of mechanisms and aetiologies of different chronic pain conditions.

## Topic #1: general guiding principles

The workshop addressed the following general guiding principles for biomarker development: first, standardized definitions; second, the process of biomarker discovery and validation; and, third, criteria for validation and evaluation of biomarkers.

According to the FDA Biomarkers, EndpointS and other Tools (BEST) glossary of biomarker terms, a biomarker is a defined characteristic that is measured as an indicator of normal or pathological biological processes, or of responses to an exposure or intervention^31,32^. Biomarkers can be based on molecular, histological, radiographic or physiological characteristics. Importantly, a biomarker is different from an end point and a clinical outcome assessment. An end point is a precisely defined variable designed to indicate an outcome of interest that is statistically analysed to address a particular research question^32^. A clinical outcome assessment is an evaluation of how an individual feels, functions or survives^32^.

The FDA^32^ and the European Medicines Agency (EMA)^33^ have also developed definitions around several categories for biomarkers (Table 1). These categories include the following types of biomarkers: susceptibility/risk, diagnostic, prognostic, pharmacodynamic/response, predictive, monitoring, safety and surrogate end points. Susceptibility/risk biomarkers identify risk factors and individuals at risk, and prognostic biomarkers can predict disease trajectory, which can guide prevention and treatment efforts and can stratify patients, thereby redefining disease categories to align with pathophysiology. Diagnostic biomarkers can confirm the presence or absence of a disease or disease subtype, and monitoring biomarkers are used to monitor disease progression, therapeutic response and safety. Pharmacodynamic/response biomarkers reflect target engagement either directly or indirectly, and predictive biomarkers can predict response to a therapeutic. Safety biomarkers reflect the potential or presence of toxicity related to a therapeutic agent. Surrogate end points are used as a substitute for a clinical end point and require extensive and robust validation^32^.

**Table 1:** Use cases for biomarkers

Type of biomarker	BEST (FDA/NIH) category	EMA category	Use cases	Examples in pain indications
Pre-incident	Susceptibility/risk	NA	Evaluate risk of developing chronic pain	Anxiety, depression^208^ *COMT* and *OPRM1* variants for the prediction of opioid efficacy and the risk of addiction^209^
Tracking and mechanism	Prognostic	Prognostic	Identify likelihood of a clinical event, disease recurrence or progression	QST for prognosis of post-surgical pain at 12 months in painful knee arthrosis^210^
	Diagnostic	Diagnostic	Identify individuals with the biologically defined disorder of interest or define a subset of the disorder (biological ‘mechanism’)	Skin biopsy and intraepidermal nerve fibre density to diagnose small-fibre neuropathy^211^ QST to identify subgroups of pain profiles in neuropathic pain conditions^212^
	Monitoring	NA	Detect a change in the degree or extent of disease over time	NA
Treatment	Predictive	Predictive	Predict which individuals will benefit from a treatment	Baseline circulating levels of the microRNA miR-548d proposed as predictive of response to intravenous ketamine in complex regional pain syndrome^93^
	NA	Enrichment	Select populations likely to benefit from a treatment	painDetect to select patients with chronic low back pain for clinical trials on the basis of nociceptive versus neuropathic pain components^213^
	Pharmacodynamic/response	Pharmacodynamic	Demonstrate a biological response to treatment; track response in biological intervention targets	TrkA phosphorylation in skin biopsies to demonstrate target engagement and inhibition of NGF–TrkA signalling^214^
	Safety	Safety signal	Indicate the presence or extent of toxicity	Joint X-ray or MRI to detect rapid progression of osteoarthritis in patients treated with antibodies against NGF
	Surrogate end point	Surrogate end point	Use as an outcome to be targeted in clinical practice or trials	NA

BEST, Biomarkers, EndpointS and other Tools; EMA, European Medicines Agency; NA, not applicable; NGF, nerve growth factor; QST, quantitative sensory testing.

### Biomarker discovery and validation

**Fig. 2:**
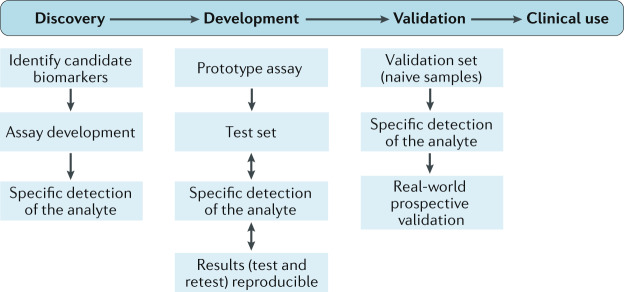
Steps to identify and develop biomarkers for clinical use. The process starts with recognition of the need for a biomarker followed by discovery of candidate biomarkers. Assay development ensues. The type of assay selected is based on the properties of the biomarker or analyte. Specific detection of the analyte is required to move forward to the assay development phase. The analyte must be measurable and the detection method must be reliable and reproducible. During development, a prototype assay is tested with a test set of samples, including both positive and negative controls. As the assay is developed, conditions are optimized, and the prototype assay is then refined, tested and retested to ensure reliable, reproducible results. For an omics assay, this process may include optimizing the pH, reducing the background signal or filtering the biological fluid to remove signal interference (for example, from haemoglobin). Once the prototype assay is optimized and produces reliable, verifiable results on test sets of samples, it must be validated using a naive sample set. Validation must be performed without knowledge of patient status to eliminate any bias in interpretation of results. If specific detection of the analyte is demonstrated, prospective validation is performed. Reproducible, reliable, sensitive and specific biomarker detection positions a biomarker for clinical use.

The biomarker development process is a systematic and directed endeavour in which the degree of validation evidence supporting the use of the biomarker increases as the intended purpose of the biomarker moves from research to clinical trials and clinical practice^34^ (Fig. 2). The process can be conceptualized as a continuum that begins with biomarker discovery and development, encompassing initial identification and preliminary proof-of-concept studies of the potential biomarker. The discovery and development phase can also include studies aimed at verifying the accuracy and reliability of the detection method, formulating a hypothesis for the context of use (COU) and testing the association between the biomarker or biomarker signature and the clinical outcomes that reflect the presence of the disease, disease prognosis, therapeutic target engagement, response to an intervention and/or potential to respond to an intervention.

Once a candidate biomarker is identified and a detection method is developed, various levels of validation are required, depending on the COU. Analytical validation involves rigorous testing of the performance characteristics of the assay or detection technology in a manner that is appropriate for the purpose of the biomarker or biomarker signature. Variables assessed during the analytical validation process include the precision, dynamic range and sensitivity of the detection method. The clinical validation step assesses the sensitivity and specificity of the biomarker or biomarker signature to identify, measure or predict the clinical outcome that it is intended to reflect. Sensitivity refers to the rate of true positive findings and specificity is associated with the rate of true negative findings. The degree of evidence that is required to provide the necessary confidence in biomarker or biomarker signature validation depends on the COU and ultimate purpose of the biomarker. As the COU moves from research use to accepted utility in clinical trials (as a marker of target engagement by a drug or response prediction for a drug) or to accepted utility in clinical practice (as a diagnostic marker or a predictor of disease trajectory to inform the course of treatment), the required degree of validation evidence increases and should include prospective, multisite validation data.

### Diagnostic and predictive accuracy

The diagnostic utility of a biomarker is directly related to its positive predictive value (PPV) and negative predictive value (NPV), which provide a ‘real-world’ assessment of true positives or true negatives, respectively, on the basis of documented prevalence of the condition in the population^35,36^. PPV and NPV are strongly influenced by both biomarker specificity and the prevalence of the condition studied. For example, in a disease that affects 1% of the population, even if sensitivity and specificity are both 98%, the PPV is only 33%. That is, a positive biomarker test only implies a 33% chance of having the underlying condition. A drop in sensitivity to 90% has little impact (PPV = 31%), but if the specificity drops to 90%, the PPV drops to 9%. Thus, testing and optimization for specificity is crucially important in biomarker development^37,38^. PPV and NPV apply to dichotomous outcomes, for example, disease present or absent. When predicting outcomes on a continuous scale, such as pain reports, the same principles regarding the importance of specificity apply to the magnitude of prediction accuracy (for example, *r*^2^). Ideally, a biomarker would predict the severity of an outcome such as pain intensity with high precision, and would not correlate with other confounding variables (for example, medication usage).

Specificity of a pain biomarker relates to its true reflection of pain or an underlying mechanism of pain. As such, it should not depend on other factors often associated with pain, for example, anxiety or depression. A biomarker for evoked pain, known as the Neurologic Pain Signature, has been tested against a range of conditions that might be confusable with physical pain^35^. Although the Neurologic Pain Signature is specific in that it does not respond to many salient, arousing affective stimuli, some classes of non-painful stimuli or mental states might activate the Neurologic Pain Signature to some degree.

### Biomarker evaluation: considerations

In addition to accuracy, specificity, PPV and NPV, there are additional important considerations. These considerations include transparency and explainability; potential for external validation; generalizability across populations, test settings and contexts; ease of deployment in clinical settings and clinical trials; procedures for ongoing validation, including positive and negative controls; and cost-effectiveness. We highlight the most important aspects of biomarker evaluation below; previous publications have discussed this process more extensively^36,37,39^. Different COUs and pain conditions are likely to require different considerations, and a single set of criteria or standards is unlikely be applicable to all situations. Beyond debates of biomarker simplicity or complexity, pain biomarkers must provide a strong enough signal for differentiating pain from non-pain conditions or successful from failed treatment, and the degree of change must be sufficiently large to rise above the clinical ‘noise’ associated with sample collection and assay variability.

#### Application of biomarkers to new settings

To enable a biomarker to be applied to new situations, standard operating procedures must be followed^36^. For example, if a composite biomarker (that is, a biosignature) consists of a combination of individual genes, proteins, MRI voxels or EEG leads, it is crucial to define precisely which variables are included and how they should be combined. For many patients, a written description of the measure will be inadequate, and electronic files defining the model or algorithm applied, along with data preprocessing and scaling steps, are essential. This process provides transparency about what the model is based on and how to derive a biomarker score for an individual test case. To date, many studies have used machine learning to develop MRI-based biomarkers for various brain disorders^39^, but only a small number of models have generated procedures that can be applied to new individuals and used for validation by other groups.

Trust that a biomarker is delivering valid predictions in a new sample or setting, such as a clinical trial of a new population, will vary as a function of the complexity of the measure and the procedures involved, and their potential failure points. Biomarkers that are simple to understand and validate, and that are biologically plausible, are likely to be more robust^39^. In addition, biomarkers will generally be more successful if the principles underlying their predictions can be explained, for example, in terms of crucial brain regions, systems, biological pathways or neurochemicals. Successful biomarkers should also have convergent validity with other methods to provide external validation that the biomarker is biologically meaningful and, for some applications, causally related to pain^40^. Validation could come from human electrophysiology, lesion studies or invasive techniques in animal studies (for example, omics, optogenetics, chemogenetics or imaging)^37^.

#### Generalizability

Inevitably, the conditions under which a biomarker is applied will differ in some ways from those under which it was developed. Therefore, biomarkers will need to generalize to conditions that differ from the original conditions^37^. Generalization can be assessed across individuals, variations in testing procedures, analysis pipelines, equipment (for example, different scanners) and populations^38^. As the test context (including the location, population, scanner, personnel and environment) will inevitably differ to some degree from the training context (such as the population and environment of the sample used for model development), diagnostic accuracy invariably decreases when applying the biomarker in new contexts, and the greater the loss in accuracy across different contexts, the less generalizable the biomarker. This factor must be taken into account in decision-making as to whether a biomarker should be further developed and validated.

#### Scaling up validation

A recommended approach to the biomarker development process is to use cross-validation in a discovery set, which can provide relatively unbiased estimates of diagnostic or predictive accuracy and adds statistical rigour (but is not infallible)^41^. Cross-validation or out-of-sample testing is a technique that is used to ensure that a model is robust if separate training and test data sets are not available. Typically, the bulk of the data are used in the training phase of creating a machine learning model and then hold-out samples (that is, the remainder of the data that are put aside for testing the model) are used to test the model. This approach is followed by further validation of the biomarker in a similar but independent population, using a similar test protocol^42,43^. Eventually, broader tests of generalization and performance across diverse populations and test protocols are performed^44,45^. This strategy scales up effort and cost in proportion to provisional success^46,47^ and establishes the boundary conditions for when a biomarker should or should not be applied.

Studies can also be designed to optimize generalizability. One such strategy is to train a model — that is, select biological features and estimate parameters — across diverse samples (for example, from different populations and ethnicities) and including diverse examples of painful and non-painful conditions. The model can thus be trained to pick out what features are related to pain in a generalizable way across these variations, but unrelated to various potentially confusable non-pain conditions^38^.

Systematic approaches to scaling up validation remain rare. For example, in our survey of machine learning-based neuroimaging biomarkers, cross-validation was used in nearly all papers, but only a small subset of studies (~9%) tested the results in an independent cohort^39^.

### Summary and recommendations

- Standard definitions regarding biomarkers, end points, clinical outcome assessments and biomarker categories that are consistent with the FDA and the EMA were conveyed and discussed by the workshop participants.
- The process of biomarker development is a systematic and highly directed endeavour, involving rigorous proof-of-concept studies, careful assessment of the performance of the detection method and an extremely rigorous multisite evaluation of the PPV and NPV of the biomarker relative to the COU.
- Cross-validation and the use of independent test sets improve the reliability and generalizability of validation data.
- The overall feasibility of the biomarker is critical to its usefulness and, therefore, should be evaluated by assessment of generalizability, explainability and validation in the real-world setting.

## Topic #2: current state of the science

A wide and growing array of biological measures, encompassing multiple domains and levels of analysis, are being explored as potential biomarkers. These measures include electrophysiology in peripheral nerves and brain; omics assays of blood, cerebrospinal fluid (CSF) and other tissues; and structural and functional imaging of peripheral tissues and the brain (Table 2). Few biomarkers based on electrophysiology, omics and imaging are currently being widely used in clinical trials or clinical practice in the pain field, although large-scale validations are beginning to emerge.

**Table 2:** Measures and assays being explored as potential pain biomarkers

Measure/assay	Peripheral nerves	Spine, joints	Soft tissue	Blood, serum and plasma	CSF and bodily fluids	Brain	Techniques	Scalability and ease of use
***Electrophysiology***								
Microneurography^215,216^	Yes	NA	NA	NA	NA	NA	Recording of spontaneous action potentials in nociceptive nerve fibres	Useful in research setting only
Nerve excitability^217^	Yes	NA	NA	NA	NA	NA	Assessment of nerve excitability (for example, threshold tracking)	Available and scaled or scalable
Slice electrophysiology (cellular recordings)^64^	NA	NA	NA	NA	NA	NA	Induced nociceptive neurons derived from human pluripotent stem cells and human tissue biopsies	Useful in research setting only
EEG^66,67,72,115,218^	NA	NA	NA	NA	NA	Yes	Laser and evoked potentials Time–frequency spectra Coherence Effective connectivity	Available and scaled or scalable
Magnetoencephalography^219–221^	NA	NA	NA	NA	NA	Yes	Laser and evoked potentials Time–frequency spectra Coherence Functional coupling	Useful in research setting only
***Bioassay***								
CGRP^222,223^	NA	NA	NA	Yes	NA	NA	Biochemistry	Specialized laboratories only
Tissue biopsy (skin punch)^51,224,225^	Yes	NA	NA	NA	NA	NA	Intraepidermal nerve fibre density	Biopsy feasible clinically, analysis requires specialized laboratory
Confocal corneal microscopy	Yes	NA	NA	NA	NA	NA	Intracorneal nerve fibre terminals	Technically easy to use, but equipment available only at specialized clinics, optometrists and ophthalmologists
***Omics***								
Genome^82,83,90,91,108^	NA^a^	NA^a^	NA^a^	Yes	NA^a^	NA^a^	Genotyping and sequencing	Available and scaled or scalable
Epigenome^93–95^	NA	NA	Yes	Yes	NA	NA	DNA methylation and microRNA arrays Sequencing Quantitative PCR MS	Specialized laboratories only, not readily scalable
Transcriptome^82,108^	NA	NA	Yes	Yes	Yes	NA	RNA sequencing Cell-free RNA	Available and scaled or scalable
Proteome^100–102^	NA	NA	Yes	Yes	Yes	NA	Antibody-based or aptamer-based MS	Available and scaled or scalable
Metabolome and lipidome^109,110^	NA	NA	Yes	Yes	Yes	NA	Gas chromatography–MS Liquid chromatography–tandem MS Nuclear magnetic resonance spectroscopy	Specialized laboratories only, not readily scalable
Immunome^97–99,105^	NA	NA	Yes	Yes	Yes	NA	Mass and flow cytometry Peripheral blood mononuclear cell stimulation assays	Available and scaled or scalable
***Imaging***								
Structural imaging (MRI, CT)^66,142,143,149,226^	Yes	Yes	NA	NA	Yes	Yes	T1-weighted scans	Available and scaled or scalable
Magnetic resonance diffusion imaging^227,228^	NA	NA	NA	NA	Yes	Yes	Tractography (white matter)	Available and scaled or scalable
Magnetic resonance elastography^111,229^	NA	Yes	NA	NA	Yes	Yes	Elastography maps	Available and scaled or scalable
Hyperspectral imaging^230^	Yes	NA	Yes	NA	NA	NA	Spectral analysis of skin or blood	Available and scaled or scalable
PET^129,223,231^	NA	NA	NA	NA	Yes	Yes	Blood flow Metabolism Neurotransmitter Microglial markers	Useful in research setting only
Functional MRI^35,114,115,134,145,148^	NA	NA	NA	NA	Yes	Yes	Stimulus-related and percept-related activation Resting-state functional connectivity Effective connectivity	Useful in research setting only
Functional near-infrared spectroscopy^232,233^	NA	NA	NA	NA	Yes	Yes	Stimulus-related and percept-related activation Resting-state functional connectivity Effective connectivity	Available and scaled or scalable
***Behaviour***								
Quantitative sensory testing^48–51,234^	NA	NA	NA	NA	NA	NA	Detection threshold and sensitivity to noxious and non-noxious stimuli Temporal summation of pain Conditioned pain modulation	Available and scaled or scalable
Facial expression^52–55^	NA	NA	NA	NA	NA	NA	Analysis of pain-related facial muscle movements	Available and scaled or scalable
Voice audio spectrum^56,57^	NA	NA	NA	NA	NA	NA	Acoustic spectrography	Available and scaled or scalable
Movement and activity^58,59^	NA	NA	NA	NA	NA	NA	Wearable devices	Available and scaled or scalable
Autonomic responses^51,235^	NA	NA	NA	NA	NA	NA	Skin conductance, pupil diameter and other physiological indicators of autonomic activity	Available and scaled or scalable

CGRP, calcitonin gene-related peptide; CSF, cerebrospinal fluid; MS, mass spectroscopy; NA, not applicable. ^a^Assumed to be tissue type-independent.

Although not strictly meeting the qualifying criteria, behavioural measures including QST^48,49^ (reviewed elsewhere^50,51^), facial expressions^52–55^, vocal characteristics^56,57^ and body movements^58,59^, as well as other data types such as geotagged measures of social and environmental exposures^60^, are often considered ‘biomarkers’. Increasingly, data within the various domains are multivariate, and analysis should probably be guided by machine learning approaches to derive robust patterns from high-dimensional and multilayered data sets^39,42^.

### Electrophysiological biomarkers

Electrophysiology can reveal pain-related electrical signals travelling from the peripheral nerves to the brain. Peripheral measures include microneurography^61–63^ and assays of induced pluripotent stem cell-derived neurons or non-neural cells^16,64^. Brain measures include EEG and related magnetoencephalography measures of evoked potentials and oscillations in brain systems associated with pain.

EEG is non-invasive, affordable and fairly easily assessed in the clinical setting. The two main types of EEG data that can be acquired are event-related potentials and resting-state EEG, which relate to stimulus-evoked and ‘spontaneous’ pain, respectively (reviewed elsewhere^65–67^). Gamma-frequency event-related potentials have been associated with pain evoked by noxious stimuli in rodents^68^ and humans^69,70^. Pain can also enhance resting-state theta (4–8 Hz) oscillations. In rat models, theta power manifests in acute, inflammatory and neuropathic pain^71^ and is reversed dose-dependently by pharmacological therapies and neuromodulation^71–75^. Theta power has been attributed to thalamocortical dysrhythmia in patients with pain^76–78^ and has been shown to be causally related to cell-specific and time-locked neural firing in the thalamus^79,80^.

### Omic-based biomarkers

Omic approaches are attractive as they provide biological markers from readily accessible body compartments. Biomarkers currently used in clinical medicine are mainly metabolites, proteins or DNA measured in blood, as blood analysis is widespread, minimally invasive and relatively cheap^81^. In the context of pain management, clinically accessible omic biomarkers have the potential to aid patient management and uncover pathophysiological mechanisms. However, such a prospect is currently anchored largely in exploratory data and will require thorough examination in large, diverse and well-phenotyped patient populations. Critical questions include whether omic signatures obtained from accessible biospecimens, such as blood, urine, CSF or exudate, reflect relevant biology, and if so, what pain conditions — for example, inflammatory versus neuropathic, or temporal patterns from acute to chronic — would benefit from such profiling.

A systems-based study published in 2017 integrated lipidomic measurements with transcriptomic profiling of lipid biosynthetic enzymes expressed in nociceptive circuits, including the skin, peripheral nerve, dorsal root ganglion and dorsal spinal cord^82^. An RNA-sequencing transcriptomics approach was used to measure the expression of biosynthetic enzymes and thereby predict the presence of lipid products in each tissue. Two previously unidentified linoleic acid-derived lipids were measured; one was elevated in blood in a human pain disorder (headache) and the other was elevated in skin in a pruritic disorder (psoriasis). In the latter case, biosynthetic expression profiles and levels of one 11-hydroxy-epoxy-octadecenoate lipid were elevated only in patients with itch symptoms. This example illustrates the potential utility of a concerted multiomic approach for biomarker discovery in peripheral tissues to advance our understanding of pain and pruritic conditions. Two other studies using a transcriptomic approach in blood also suggest that gene expression signatures related to painful conditions can be identified in peripheral tissues^19,83^. However, further studies are required to extract and validate an actionable biomarker from these analyses.

Substantial efforts in other medical fields, including neuropsychiatry, provide additional reasons for cautious optimism regarding the utility of omic biosignatures in accessible tissues. A prominent example is the use of genotypic and proteomic features as prognostic and diagnostic biomarkers in Alzheimer disease (AD). The carrier status of the apolipoprotein E ε4 allele allows patient stratification, as carriers of two copies of this allele have a 12-fold increased risk of developing AD^84^. CSF levels of amyloid-β_42_, total tau and hyperphosphorylated tau are recognized diagnostic biomarkers for observational and interventional clinical trials^85,86^. Notably, a longitudinal study in >1,500 individuals suggested that the plasma level of the axonal protein neurofilament light is a useful biomarker for tracking disease progression and treatment responses in patients with AD^87^.

Important developments in the omics field include the recognition that prevalent pain conditions are likely to be polygenic in nature, which might explain why single gene variants often account for small effect sizes that fail replication^88–90^. Derivation of polygenic pain signatures in large patient cohorts has been proposed as a crucial next step to identify robust pain biomarkers^91^.

Clinical epigenetics is an evolving field strongly rooted in oncology, where cell-free DNA methylation and microRNA signatures in plasma show promise as diagnostic and prognostic markers^92^. In pain research, early evidence suggests that circulating microRNAs can allow profiling of chronic pain conditions and prediction of treatment responses^93–96^. Considering the substantial crosstalk between the immune and nervous systems, systemic proteomic and cell-based signatures might mirror biological aspects that are pertinent to the initiation and maintenance of pain^97–99^. Novel antibody-based and aptamer-based proteomic platforms measuring >1,000 proteins are promising discovery tools that have uncovered predictive signatures in cardiovascular and feto-maternal medicine^100–103^. Novel high-dimensional and single-cell assays, including mass cytometry-derived immune signatures in peripheral blood, have predicted the resolution of post-surgical pain^104–106^. More conventional functional assays in peripheral blood mononuclear cells revealed strong signatures in patients with fibromyalgia^107^. Many of these techniques can be augmented by RNA-sequencing approaches, which provide high-resolution, comprehensive and quantitative expression profiling of basal-state and induced genes^82,108^. Metabolomic approaches are also appealing, as metabolites are the final downstream products of translation and, as such, are close to a studied phenotype^109^. However, few studies have examined metabolomics profiles in patients with pain, and signatures derived in other fields, such as cardiovascular medicine and neurology, remain preliminary^81,110^.

### Imaging-based biomarkers

Neuroimaging can reveal structural and functional abnormalities in pathways directly and indirectly related to nociception, pain and other aspects of function. Ultrasound, MRI and CT have broad applicability for the detection of gross structural pathology in peripheral and central tissues. Emerging peripheral measures include magnetic resonance elastography for detection of shearing forces in tissue^111^ and hyperspectral imaging to non-invasively detect cellular and biochemical changes in skin, blood and other tissues^112,113^. The latter technique can be cross-linked with omics. An important use of imaging is to study peripheral nerves, CNS pathways and brain networks in relation to self-reports of pain^1,48,91,114–116^ and co-occurring aspects of function and well-being, such as fatigue, fear and anxiety, and depression. Preclinical imaging studies have identified potential pathophysiological pain mechanisms in multiple nervous system pathways, including sensitization of nociceptive neurons throughout the neuraxis^23,79,91,114,117^, alterations in frontostriatal pathways^71,106,117^ and many others^118–120^. In some cases, brain changes potentiate descending pain facilitation, thereby amplifying spinal cord responses to noxious events^121,122^.

Identification of abnormalities and functional sensitivity in brain pathways could provide a basis for studying and grouping patients with chronic pain on the basis of brain alterations. Neuroimaging is also increasing our understanding of the role of the spinal cord and brainstem systems in acute and chronic pain. Currently, efforts are focused on simultaneously imaging the entire CNS to better characterize ascending and descending nociceptive and pain modulatory systems^123–125^. Characterization of CNS function and abnormalities will help us to understand the development and maintenance of chronic pain. CNS abnormalities might cause or maintain pain independently of or in interaction with peripheral alterations, thereby complementing more traditional measures of pain.

Common forms of structural imaging include anatomical MRI to measure cortical thickness, volume and grey matter density, and diffusion-weighted imaging to measure white matter integrity and pathways^126^. Functional imaging can be accomplished with PET and functional MRI (fMRI)^126,127^. PET can measure metabolism and neurochemistry of hundreds of neurotransmitters and neuropeptides in the resting state and during tasks or stimulation. fMRI can measure resting functional connectivity and task-evoked or stimulation-evoked activity and connectivity. Magnetic resonance spectroscopy can be used to measure resting or task-evoked levels of metabolites, such as glutamate and GABA, although the spatial and temporal resolution is low. PET has been used for decades in preclinical and clinical drug discovery research to image drug penetrance and pharmacodynamic effects^128^, and various PET radioligands can be used to image brain neuropeptides (for example, opioids)^129,130^, neuroinflammation^131,132^ and peripheral immune activity^133^ in relation to pain.

Current work is focused on developing and validating fMRI-based measures as biomarkers for evoked pain sensitivity^35,44,134,135^ and as diagnostic^136–143^, prognostic^116,144,145^, predictive^146–149^ or response^120^ biomarkers for clinical pain. Several reviews provide more detailed coverage of neuroimaging-related biomarker development^38,39,91^ and emerging biomarkers for clinical pain^114,150^.

### Composite biomarkers

At present, we have no universally accepted composite assessment for chronic pain that incorporates biological and behavioural measures to arrive at a valid and comprehensive measurement of the pain experience. The current standard for assessment is primarily the patient’s self-report of pain intensity and severity^151^. Patient report has been shown to correlate with brain activity and patterns of brain functional connectivity^44,139,152^. A comprehensive and quantitative assessment of pain correlates, incorporating the latest findings regarding pain biomarkers (for example, fMRI and/or omics-derived), behavioural evidence (for example, facial expressions) and physiological measures (for example, heart rate variability), might augment the validity and meaning of self-report. Subsequently, a comprehensive and composite assessment constituting a ‘chronic pain signature’ may lead to new and improved treatments. However, chronic pain signatures could vary depending on the context; for example, a diagnostic chronic pain signature may be different from a prognostic chronic pain signature or from a predictive chronic pain signature. Nonetheless, identification of biomarkers that reflect pain mechanisms could enable the development of targeted treatments^91^.

Systems-level biological profiling of pain in clinical settings requires a harmonized set of omics and brain assays, targeting and linking multiple levels of analysis^101^. These brain assays may include brain imaging (fMRI or PET), as discussed above, fluid (blood or CSF) levels of CNS proteins or detection of mutations in genes encoding pain-associated molecules, such as voltage-gated sodium channels. The systematic analysis of the large amount of data produced by each of these high-throughput assays will lend itself to greater use of specialized machine learning tools in the future^101^. When applied to pain, higher-order integration of such data sets in a ‘multiomics’ setting will rely on complex machine learning pipelines that remain robust despite inconsistencies in the intrinsic properties of these high-throughput assays, as well as cohort-specific variations. Signal strength must be optimized to reduce noise associated with clinical and technical variables such as patient presentation and sampling procedures. Addressing these challenges necessitates close collaborations between consortia to develop well-curated biopsy and body fluid collections, harmonized data sets and analysis pipelines, and large-scale machine learning systems^153–155^. Importantly, algorithms that are developed should have the capacity to integrate non-biological measurements, including behavioural, clinical and imaging data^156,157^.

To enable true clinical impact, these computational approaches need to move beyond predictive power generated by high-dimensional and multilayer models. ‘Explainable’ artificial intelligence is currently an active area of research to make complex models understandable by human investigators^158^. Such algorithms will facilitate downstream validation, improve mechanistic and biological understanding, and enable the development of scalable assays to capture robust effect sizes in resource-limited and regulatory settings^159,160^.

### End points and clinical outcomes

A clinical end point is a defined variable that is intended to reflect an outcome of interest in a clinical trial. End-point specifications typically include the type and timing of assessments and the assessment tools used, and possibly other details, such as how multiple assessments within an individual are to be combined^32,161^. A clinical outcome directly reflects how a patient feels, functions or survives. Clinical outcome assessments can be made through report by a clinician, a patient, a non-clinician observer or a performance-based assessment; these clinical outcome assessments are termed clinician-reported, patient-reported, observer-reported and performance outcomes, respectively. A surrogate end point indirectly predicts clinical benefit or harm on the basis of epidemiological, therapeutic, pathophysiological or other scientific evidence^32,161^.

The Initiative on Methods, Measurement, and Pain Assessment in Clinical Trials (IMMPACT) has recommended multiple core outcome domains^162^. Measures of pain severity, pain qualities and physical functioning are most often designated as primary end points in pain trials. The Numerical Rating Scale measures of pain intensity, in the form of single-item 11-point (0–10) scales, the 10-cm Visual Analogue Scale and multi-item measures, such as the Brief Pain Inventory, are well validated, widely used and generally considered the gold standard for pain intensity outcomes^22,163^. For patients who are unable to provide numerical ratings, validated pictorial and pain behaviour-based assessment tools are available^24,25^.

Intensity is only one dimension of pain, and instruments such as the McGill Pain Questionnaire can measure numerous qualities of pain (for example, burning versus aching) and affect (for example, discomforting versus excruciating)^23^. The Neuropathic Pain Symptom Inventory, painDETECT, DN4 and the Leeds Assessment of Neuropathic Symptoms and Signs (LANSS) questionnaire are convenient, brief and well-validated tools to assess neuropathic pain^30,164,165^. Some evidence has suggested that different pain qualities respond differentially to specific neuropathic pain treatments^166^. Despite their broad use, self-report-based measures are highly variable and only modestly reliable, even with intensive training programmes designed to improve pain reporting accuracy^27^, highlighting the urgent need to identify pain biomarkers. Measures of pain sensitivity and modulation, assessed using QST, might serve as important biomarkers of phenotypes and outcome measures to help identify specific mechanisms that are influenced by treatment^163,167^.

Patient-assessed measures of pain are generally supplemented by additional end points. An IMMPACT survey revealed that patients consider physical functioning, enjoyment of life, emotional well-being, sleep and fatigue to be important outcome domains^168^. Physical functioning in various arenas, including work participation and recreation, is adversely affected by chronic pain. As a treatment end point, physical functioning can be measured by patient self-report (for example, measures such as the Short Form-36 physical functioning scale, the PROMIS (Patient-Reported Outcomes Measurement Information System) and the Pain Disability Index), performance-based metrics and objective measures of activity^169^. Emotional functioning, typically measured by patient report of measures of affect, both general (for example, depression and anxiety) and pain-specific (for example, catastrophizing and fear of pain), is widely used as a secondary end point^170^.

Global ratings of improvement and satisfaction provide an opportunity for participants to aggregate their experience into one overall measure of the treatment’s effects^162^. For example, the Patient Global Impression of Change scale is a well-validated single-item rating of treatment-related improvement on a 7-point scale ranging from ‘very much improved’ to ‘very much worse’^171^. In addition, measures of adverse effects and events are an essential component of all clinical trials, as they are a primary reason for trial attrition and have detrimental effects on function and quality of life^172^. Participant disposition — for example, adherence to treatment and reasons for premature withdrawal — is an additional core outcome measure^162^. Collectively, these multidimensional clinical end points reflect the broad biopsychosocial nature of the pain experience and cover numerous specific domains in which pain treatments can provide clinical benefits.

### Summary and recommendations

- Complex physiological biomarkers, such as QST, facial expression, vocal characteristics and body movements, have become an expanding area of biomarker research. Machine learning approaches to analyse these data are under development to derive robust patterns from high-dimensional and multilayered data sets^39,42^.
- Electrophysiological pain biomarkers, such as EEG, are non-invasive and can have a high level of utility, but are likely to require the same types of analysis as the complex physiological biomarkers.
- Omics biomarkers for pain are also becoming increasingly complex and multidimensional because of the crosstalk between immune and neural circuitry. As a result, an increasing number of these biomarkers are biosignatures rather than single-molecule biomarkers.
- Imaging biomarkers for pain utilize multiple types of imaging, including MRI, ultrasound, fMRI and PET. These biomarkers can reflect circuitry and pathways in the pathophysiological state, providing complex anatomical signatures that could be combined with omics biomarkers to form multimodal signatures of chronic pain with and without treatment.
- Composite biomarkers for pain can include imaging, omics and physiological measures, the precise nature of which could depend on the COU (that is, diagnostic versus prognostic). These complex measures will require algorithms to simplify final end points that are explainable.
- Explainable artificial intelligence is a new area that may make the complex models described above more feasible for broad use by the clinical and research community.
- Many self-report pain outcome measures are available, but they are highly variable and only modestly reliable, highlighting the need for objective pain biomarkers.
- QST is used as a pain phenotype biomarker to identify mechanisms of pain, but requires additional validation and optimization.

## Topic #3: implementing pain biomarkers

The use of a biomarker in treatment development or clinical practice requires a clear understanding of the pain construct assessed by that marker (Table 3). A binary diagnostic marker may be useful for the purpose of disease classification, whereas qualitative grading scales or quantitative methods will be necessary to evaluate the activity of pain mechanisms or treatment response. Rigorous evaluation of construct validity, assay sensitivity and reliability may entail collecting evidence through preclinical studies and clinical trials, the latter potentially involving healthy volunteers and patients.

**Table 3:** Potential pain biomarkers used in clinical trials

Pain disease state	Biomarker	Correlation to disease	Correlation with pharmacodynamic outcome	Correlation with pain state	Clinical efficacy shown
Rheumatoid arthritis and neuropathic pain	CCL concentration in cerebrospinal fluid and plasma	CCL in neuropathic pain	Highly efficient antagonism of CCR2	No	No^236,237^
Inflammatory pain	TRPV expression	TRPV elevated	TRPV antagonism leads to reduction in inflammation	Yes	No^238,239^
Chronic back pain	Nerve growth factor	High	High	Yes	Yes^240^
Migraine	CGRP concentration	Elevated in disease state	Yes	Yes	Yes^241^
Neuropathic pain	Resting-state functional connectivity, temporal summation of pain	No specific correlation	Unknown	Yes	Yes^148^
Painful diabetic neuropathy	Conditioned pain modulation	No specific correlation	Yes	Yes	Yes^242^
Migraine, fibromyalgia (nociplastic pain)	Conditioned pain modulation	Poor conditioned pain modulation capacity	Yes	Yes	Yes^183,243–245^

CCL, CC-chemokine ligand; CCR2, CC-chemokine receptor 2; CGRP, calcitonin gene-related peptide; TRPV, transient receptor potential cation channel subfamily V.

One example that illustrates the complexity of construct validity in painful neuropathies is the use of skin punch biopsies to determine the density of intraepidermal nerve fibres (IENFs)^173^. Standardized histological quantification and determination of age-specific normal values have helped to establish IENF density as a sensitive indicator of peripheral neuropathies involving thinly myelinated or unmyelinated (small-diameter) nociceptive nerve fibres (known as Aδ and C fibres, respectively)^51^. Conditions that selectively affect these fibres are termed small-fibre neuropathies^174^. Pain is a prominent clinical feature of small-fibre neuropathies. However, its correlation with the loss of IENF is uncertain^175,176^. Consequently, IENF density may serve as a histopathological biomarker of nociceptive nerve fibre involvement in a peripheral neuropathy but does not reliably indicate the functional consequences, that is, the presence of spontaneous pain or abnormal sensitivity to painful stimuli. The dynamic nature of pain mechanisms is another challenge for the use of biomarkers. Inflammatory and neuropathic processes are not static^177^, and their relative contributions to clinical pain disorders can vary over time. Capturing these changes through repeated testing may be difficult in clinical practice if the diagnostic procedure is invasive, poorly tolerated by patients or costly. Inclusion of biomarkers in a clinical trial increases the complexity of the scientific and statistical design, and adds to the operational challenges of trial execution and data collection. Such an investment requires a thorough understanding of the diagnostic or therapeutic value to be gained. From a treatment developer’s point of view, markers of pain mechanisms support patient stratification in a more meaningful way than pain intensity, the emotional response to pain or comorbidity^177,178^. A sensitive marker of target engagement that reliably predicts pain reduction might allow early identification of treatment responders, enable a streamlined trial design and increase the probability of success. The net impact of a biomarker assay further depends on its biological specificity and robustness against placebo effects^179^. Biomarkers will be most useful if they deliver proof of target engagement or biology, demonstrate the validity of a therapeutic concept or help identify treatment responders.

Predictive biomarkers that qualify as surrogate end points may help to shorten trial duration during treatment development and expedite the regulatory approval of effective pain treatments. Biomarkers meeting some of these criteria, including measures of thermal or capsaicin-evoked transient receptor potential ion channel activity^180,181^ or microneurographic recordings of nociceptive fibre firing^182^, have been employed in phase I and II trials of emerging pain treatments (Table 3). Inclusion of biomarkers in a phase III trial designed to obtain regulatory approval requires robust evidence that the biomarker is not only relevant for disease but is also sensitive to change by the intervention^183^. The potential to achieve greater effect sizes by targeting the treatment to a well-defined subpopulation of patients with a substantial unmet need could justify the effort required to generate such evidence. However, early interaction with regulatory authorities (Box 2) will be necessary to gauge the implications of biomarker use for the development path of a product and the indications covered in the product label.

Regulatory monitoring of biomarker use will help maintain rigorous quality standards that are compliant with Good Clinical Practice. Accessibility of biomarkers for pain specialists as well as general practitioners with limited scientific, technical and financial resources also needs to be considered early in the biomarker development process. Successful biomarker discovery and implementation requires reliable clinical evidence (the standard for what is being measured) and an accurate assay technique (specificity of what is being measured).

Finally, the process of biomarker discovery and validation is complex and requires multiple areas of expertise in a collaborative or team approach. Transparency and data sharing are important factors in facilitating efficient pain biomarker development. Implementing biomarkers for diagnosis, prognostic prediction, patient stratification or monitoring of treatment response can also benefit from public–private partnership collaborations, as exemplified by the Alzheimer’s Disease Neuroimaging Initiative (ADNI).

Box 2 Important regulatory agencies The following agencies have ongoing active collaborations with regard to biomarker development guidance:

- European Medicines Agency (EMA): https://www.ema.europa.eu/en
- US Food and Drug Administration (FDA): https://www.fda.gov/
- Health Canada: https://www.canada.ca/en/health-canada.html
- The Japanese Pharmaceuticals and Medical Device Agency (PMDA): https://www.pmda.go.jp/english/index.html
- National Medical Products Administration — formerly the China Food and Drug Administration (CFDA): https://www.emergobyul.com/resources/china/china-food-drug-administration

### Summary and recommendations

- Both categorical and quantitative pain biomarkers are necessary, depending on the COU for the biomarker. Different design and analysis strategies are required for these two types of biomarkers.
- Rigorous validation of a biomarker (construct validity, detection method validation and clinical validation) is extremely time-consuming and may not match the timelines for clinical trial use. It is important to begin developing biomarkers early in the drug development process.
- The dynamic nature of pain adds complexity to the development and validation of pain biomarkers.
- The use of surrogate end points for pain could substantially improve clinical trial design and increase the efficiency of clinical trials, but validation requirements are very high for surrogate end points from a regulatory standpoint. Therefore, interaction with regulatory officials should occur early in the drug development process.
- A collaborative approach to use of pain biomarkers in clinical trials is recommended, whereby academic groups, patients, regulatory agencies and biopharmaceutical entities work together to develop pain therapeutics and their associated biomarkers.
- Transparency and data sharing are vital to a more efficient and effective approach to biomarker and end point development.

## Topic #4: role of regulatory agencies

Several biomarkers related to pain have been evaluated by regulatory agencies (Box 2; Table 4). Current practices for quantitatively measuring pain intensity and pain relief (for example, reduction in pain intensity) include the 0–10 Numerical Rating Scale or the Visual Analogue Scale, in combination with an assessment of health-related quality of life. These outcome measures form the basis of clinical development guidelines for acute, chronic, neuropathic and other specific pain syndromes in the United States and Europe. At the time of writing, the FDA is about to release new guidelines on analgesic drug development^184^ to facilitate the evaluation of alternatives to opioids to help combat the opioid epidemic. A similar goal is being pursued by Health Canada^185,186^. The current and proposed new FDA guidelines for drug development in pain do not specifically address biomarker evaluation. However, this issue is addressed by numerous other publications and initiatives, including ICH E16 (ref.^187^), the FDA Biomarker Qualification Program, which features a draft FDA guidance document on the evidentiary framework for biomarker development^34^, the FDA Drug Development Tool Qualification Programs and an EMA document entitled ‘Essential considerations for successful qualification of novel methodologies’^188^.

**Table 4:** Examples of biomarkers evaluated by regulatory agencies

Biomarker/tool	What does it do?	Patient stratification	Surrogate end point for pain
Quantitative sensory testing profile in neuropathic pain	Somatosensory phenotype profiling	Patient stratification by phenotyping sensory profile	Supportive of evoked pain rating but does not evaluate spontaneous pain^190^
Microneurography	Measuring spontaneous C-fibre activity	Yes, in laboratory setting	In early trials, C-fibre activity correlated with pain intensity — more trial data requested^190^
Confocal corneal microscopy	Non-invasive diagnostic measure of peripheral small-fibre neuropathy	Yes, for small-fibre neuropathy in diabetes	No, accepted for diabetic neuropathy only^190,246^
Skin biopsy — nerve fibre density	Diagnosis of nerve injury	Yes, would be approved if used	No
14-3-3η	Diagnostic for rheumatoid arthritis, differentiation to osteoarthritis	Patient selection for clinical trials	No

Genomics have been accepted for patient stratification or definition of patient populations. Imaging or electrophysiology for quantification of pain modulatory systems has not yet been subject to regulatory evaluation. Few fluid biomarkers have yet been specifically evaluated in relation to pain.

### Current regulatory guidance

Patient assessment of perceived pain intensity should continue to be the primary end point in a clinical trial. Biomarkers can serve as a supportive secondary measure or, in early-phase clinical trials (phase I and II), as a pharmacodynamic marker or surrogate end point. Similar to other therapeutic areas, regulatory authorities view biomarkers in pain drug development as diagnostic tools for patient stratification, cohort enrichment and development of eligibility criteria for inclusion in a drug trial. Biomarkers are also viewed as useful prognostic tools and/or surrogate end points. For a biomarker to qualify for use in these contexts, its clinical utility in terms of guiding diagnostics, patient management and outcomes needs to be demonstrated in a rigorous fashion. In addition, biomarker performance — that is, sensitivity, specificity and robustness — and the analytical platform must fulfil regulatory criteria. For composite biomarkers (diagnostic or outcome), the performance criteria must be characterized both individually and in combination. At this time, no surrogate end points for pain are qualified by the FDA for use in clinical trials^189^.

In 2015, the private–public partnership Innovative Medicines Initiative (IMI) Europain put forward QST as a potential diagnostic and stratification tool in neuropathic pain trials. On the basis of the scientific evidence put forward, the EMA concluded that QST was acceptable to use as a stratification tool in clinical trials for peripheral neuropathic pain, provided that differential outcomes according to QST sensory phenotype could be confirmed through replication in a clinical trial. If this goal was accomplished, the indication of use would be limited to the subpopulations studied; for example, a trial in peripheral neuropathic pain, where patients were included on the basis of their sensory profile, would be acceptable. QST variables have also been accepted as secondary end points^190^.

Electrophysiological measurements (spontaneous C-fibre activity) have been generally thought useful to confirm the origin of neuropathic pain, but confirmatory trials were deemed necessary before they could be considered a surrogate efficacy end point.

In addition, there is an opportunity to have companion diagnostic biomarkers for a given drug treatment, which could facilitate the personalization of therapeutics. One example is a blood biomarker test that would indicate whether a patient is likely to respond to a certain medication based on a specific pain condition. A list of early biomarkers being considered by regulatory agencies is provided in Table 3.

### Summary and recommendations

- Regulatory agencies regard biomarkers as adjunct tools for use in clinical trial design by acting as indicators of drug target engagement, tools for patient stratification or therapeutic response predictors.
- Rigorous validation is required for the use of biomarkers as adjunct end points in clinical trials.
- No FDA-qualified surrogate end points for pain therapeutics are available at this time.
- Regulatory agencies in the United States and Europe are actively involved in facilitating best practices for biomarker validation.
- The EMA has concluded that QST will be acceptable to use as a stratification tool in clinical trials for peripheral neuropathic pain if differential outcomes according to the QST sensory phenotype can be confirmed through replication in a clinical trial.

## Topic #5: societal and ethical issues

As highlighted above, pain is currently defined by patient self-report^1,2^. However, objective pain biomarkers might be useful in individuals who are unable to effectively communicate pain, including infants^191–194^, minimally conscious patients^195,196^ and people with dementia^197^ or other intellectual disabilities^55,198–200^. The inability to report pain can result in continuing harm to such vulnerable groups. For example, US courts often will not take action unless physical evidence of pain or mistreatment is provided. Even when patients can report pain, clinicians’ mistrust of reports can have similar adverse effects. These harms are exacerbated by mismatches between pain communication norms across cultures^201–203^. In addition to their potential role in cases where self-report of pain is not possible, objective pain biomarkers might help us to determine the level of response to a treatment, predict who will develop chronic pain, identify and test novel mechanistic targets for treatments and select personalized treatment. Objective, accurate, verified pain biomarkers may also be useful in support of disability insurance or legal claims^3^. However, no universally accepted objective pain biomarker currently exists.

Undertreatment or overtreatment of chronic pain can result in physical, emotional, social or financial harm to patients, and misuse of biomarkers can also be harmful. For example, a false-negative result could disqualify a patient from the right treatment, and a false-positive test result could subject a patient to the risk of a potentially harmful and/or ineffective treatment. The issue of false-positive test results was recently highlighted by the use of prostate-specific antigen (PSA) screening for prostate cancer. Until 2008, professional organizations recommended yearly PSA screening for men beginning at 50 years of age. However, more recent research found that false positives of this biomarker were leading to substantial overtreatment of men and exposing them to unnecessary surgery and radiation therapy^204^.

An international taskforce reviewed the status of the utility of brain imaging for the diagnosis of chronic pain^37^ and also highlighted how its misuse can have ethical and societal implications^37^. Brain imaging is currently used as a tool to understand pain mechanisms, and the taskforce recommended standards for neuroimaging to meet in order to be applied as a pain biomarker for clinical diagnosis^37^. Although it has already been introduced in litigation, brain imaging has not yet met the criteria to incontestably support or dispute a legal claim of chronic pain; however, it might augment patient self-report^4^.

Liquid biomarkers, such as DNA, RNA, proteins and metabolites, also have societal and ethical implications. Genetic testing carries risks relating to insurability, employment, stigmatization and law enforcement, both for the affected individual and their family members. For, example a biomarker predicting a future debilitating pain condition that could be financially costly to insure could limit both future employment and employability. Employers make an investment in employees through education and training. In addition, health insurance packages are an incentive offered by some employers to attract and keep valuable employees. The employer’s cost to insure employees increases in parallel with the use of healthcare resources by the group. Even if insurance is not provided by an employer, a debilitating pain condition resulting in increased usage of healthcare resources, absenteeism and lost productivity, either through decreased efficiency or complete removal from the workforce, is a loss to the employer. Thus, an employer is less likely to hire someone with a known future debilitating pain condition.

Healthcare and insurance providers must find a balance between patient privacy and disclosure of information. The United Nations International Declaration on Human Genetic Data supports protection of data privacy and security, and the Genetic Information Nondiscrimination Act was enacted to protect individuals from employment and health insurance discrimination^5^. Genetic testing is recognized to carry risks but also has societal benefits. For instance, genetic testing may identify a mutation that provides an explanation for the pain condition and leads to optimized pain management. Accurate diagnosis and pain management assist with appropriate resource utilization, thereby benefiting society^205,206^. Imaging and genetic testing both share issues related to patient privacy or potential future harms (for example, loss of insurability or employment). Potential misuse of data or loss of privacy through the use of imaging with artificial intelligence or machine learning algorithms poses risks to both society and individual patients. Harms could become generalized to the larger familial or ethnic group if a pain condition is determined to be of genetic origin.

Chronic pain disorders are classified primarily on the basis of anatomical location or the relationship to an underlying disease or injury^7–11^. These pain states may or may not generalize to other pain conditions. For example, the mechanisms and biomarkers associated with the condition of chronic low back pain might not be the same as for chronic migraine. Therefore, pain may manifest differently, and treatments and treatment responses could vary across pain conditions. Furthermore, the nature of chronic pain, for example, neuropathic or non-neuropathic pain, influences the response to treatment, healthcare utilization and quality of life^12^. As an important crucial step towards better classification of chronic pain conditions, the new WHO International Classification of Disease (ICD-11) has adopted the IASP-developed classification system, with pain receiving its own improved classification and diagnostic coding^1^. Without the systematic classification of pain, the resources utilized for pain treatment and loss of productivity due to pain conditions are not measurable. Society requires a method of measuring resource consumption to ensure access to care^1^.

Research has identified multiple mechanisms that contribute to the development or maintenance of chronic pain; however, these mechanisms are not fully understood^13^. Moreover, there is considerable heterogeneity within and across subgroups for each chronic pain condition (as currently classified), and the underlying pathophysiology of this variability is not fully understood. Understanding the underlying pathophysiological mechanisms may be the optimal way to select appropriate treatment. Biomarkers need to identify specific mechanisms and subgroups of patients within or across chronic pain disorders.

Objective biomarkers may validate a patient’s self-report, but should not confer distrust^14–17^. Because chronic pain is a complex biopsychosocial disorder, biomarkers might have limited use in determining the severity, impact and disability related to an individual’s pain condition. Also, structural findings often correlate poorly with pain severity. For each biomarker, a specific COU must be narrowly defined and broadly understood, so as to prevent potential misuse, inappropriate conclusions and bias. As each biomarker is developed and validated for a specific COU, use outside that COU could constitute misuse.

Socio-economic and racial factors contribute to disparities in both the experience of chronic pain and pain care, with poorer neighbourhood socio-economic status and black race being associated with worse outcomes^18,19^. Inaccessibility of primary and specialty pain care, lack of conveniently located pharmacies or therapists, language barriers or culturally subscribed communication styles of both patients and providers, and varied life experiences all contribute to suboptimal pain management^19,20^. Communication with technical medical jargon is culturally appropriate (subscribed) within healthcare provider groups, but when speaking with a patient is likely to be ineffective. Likewise, a person’s culture influences how they communicate pain, for example, whether they are stoic or emotive. Such personality traits could influence a patient’s self-assigned pain intensity score, resulting in inadequate or inappropriate treatment^207^. If biomarkers for pain are to contribute to pain diagnosis, management and development of new therapeutics, they must be tested on and applicable to diverse populations^3,4,14–17,21–29^.

### Summary and recommendations

- Objective, accurate, verified pain biomarkers might be useful in support of disability insurance or legal claims.
- Biomarkers could carry risks relating to insurability, employment, stigmatization and law enforcement, for both the affected individual and their family members, emphasizing the importance of very rigorous validation before use.
- Systematic and approved classification of pain is a necessary adjunct to measures of pain.
- Translation of pain measurement to clinical practice requires effective communication and sensitivity to the importance of environmental influences on the patient’s perception of pain.

## Conclusions

Pain can be conceptualized as a conscious interpretation of a sensory stimulus that activates nociceptive afferents and the mental projection of that stimulus onto a body part. Often, the stimulus–pain relationship can be weak, distorted or absent, such as in many chronic pain conditions. Pain assessment is the process of approximating a person’s self-narrative or ground truth, and pain biomarkers can aid this effort. In addition to representing pain intensity, biomarkers can identify the factors that predict treatment outcomes. The general consensus among the authors of this paper is that there is a substantial unmet need for better biomarkers to facilitate the development of non-addictive pain therapeutics for the following reasons. First, biomarkers can provide an indication, either directly or indirectly, that a therapeutic intervention reached its intended molecular target. Second, biomarkers can predict the response to a therapeutic. Third, biomarkers can improve the quality of a clinical design by allowing the stratification of patients into specific subcategories of a disease or condition. Last, biomarkers can be used to monitor safety and efficacy over time^16^. In addition, we agree that biomarkers, signatures and clinical end points have the potential to transform the medical landscape by introducing pain diagnostic tools that are precise, fast and adaptive to an individual’s state, hence radically changing how pain is managed, monitored and treated on an individual basis. Machine learning techniques can rapidly interpret patterns in reams of data, and thus identify key information that healthcare providers cannot resolve in a brief patient visit at the point of care. The addition of biomarkers with a mechanistic relationship to the pain may lead to novel non-addictive therapeutics.

The emphasis on composite biomarkers and end points is likely to grow, given the widespread acceptance of the complexity and multidimensionality of the pain experience. In addition, due to the large variability and heterogeneity in pain conditions and sensitivity among individuals, validation should be conducted using personalized data sets and correlative designs that could facilitate flexible use of the biomarker or signature for both categorical and personalized classifications. New technologies are providing composite, functional representations of neural circuits that could potentially predict pain transition to a chronic state or reflect abnormal sensitivity. However, multiple technical challenges hinder the development of portable diagnostics and adaptive closed-loop therapies, including dimensionality reduction of neural data, standardization of artefact removal in imaging and electrophysiological data, and miniaturization of wearable sensors and high-speed processors. In addition, ethical issues such as verification of a patient’s self-report by healthcare providers, withholding treatment and the legal ramifications of discrimination and incrimination are serious societal challenges that must be considered when developing biomarkers for pain.

We believe that this is an opportune time to discover, develop and validate new pain biomarkers, as compelling scientific knowledge can be gained in the process. In theory, a strong biological rationale exists to develop pain biomarkers that rely on brain signals recorded non-invasively. In practice, however, linking specific patterns of neural activity to distinct mental states is the ‘hard problem’ in neuroscience. Elucidating the neural circuits of the multiple pain dimensions will require ultra-fast recording techniques, robust computing power and big data analytics, as well as fine control of neural elements to demonstrate causality, for example, using optogenetics.

As the volume of biomarker data continues to grow, artificial intelligence-driven scientific discovery is emerging complementary to the more traditional hypothesis-driven approach. In some cases, machine learning algorithms can be leveraged to rank the features that contribute to the accuracy of an algorithm’s prediction of pain states, thus generating novel and testable hypotheses. Additional important considerations to better align diagnosis with therapy include demonstration of a biological rationale, standardization of protocols and equipment, cross-validation in large-scale, multicentre trials controlling for age, gender and culture variables, defining end points and use cases, cost-effectiveness and ease of use.

It is important to recognize the need for a detailed and rigorous path towards discovery and validation of pain biomarkers in research and clinical settings. The workshop participants agreed on the importance of standard definitions and a clear process to validate and develop first-generation empirical, scalable and translational biomarkers that complement the patient’s self-report and obviate the need for inconclusive and costly diagnostic tests. A risk–benefit analysis for the multiple types of pain biomarkers is still needed in light of unprecedented levels of societal and economic pressures urgently calling for alternatives to current gold standards for pain assessment.

Biomarker development for pain is still in its early stages. However, substantial increases in funding and resources for biomarker and non-addictive therapeutic development, resulting from initiatives such as the EU–IMI Europain consortium and the NIH HEAL Initiative, should stimulate research and technology development in this area of high unmet medical need.

## Glossary

Target engagement: A demonstration that the drug in question reaches its target in the body and, in doing so, results in a measurable effect (for example, opioid binding to the μ receptor).
Quantitative sensory testing: (QST). A series of standardized psychophysical protocols that are used to quantify sensory function.
Context of use: (COU). A statement that fully and clearly describes the way the medical product development tool is to be used and the medical product development-related purpose of the use.
Sensitivity: The proportion of true (actual) positive findings that are correctly identified, also known as the true positive rate or probability of detection.
Specificity: The proportion of true (actual) negative findings that are correctly identified, also known as the true negative rate.
Positive predictive value: (PPV). The probability that people with a positive screening test result do indeed have the condition of interest, taking into account the prevalence of the disease or condition.
Negative predictive value: (NPV). The probability that people with a negative screening test result do not have the condition of interest, taking into account the prevalence of the disease or condition.
Transparency: Electronic files define the model or algorithm applied, along with data preprocessing and scaling steps for complex biomarkers with algorithm end points.
Explainability: The ease of understanding an algorithm or composite biomarker and its application.
Dimensionality reduction: Reducing the number of random variables to a smaller set of principal variables.
